# Swine Influenza Virus (H1N2) Characterization and Transmission in Ferrets, Chile

**DOI:** 10.3201/eid2302.161374

**Published:** 2017-02

**Authors:** Nicolás Bravo-Vasquez, Erik A. Karlsson, Pedro Jimenez-Bluhm, Victoria Meliopoulos, Bryan Kaplan, Shauna Marvin, Valerie Cortez, Pamela Freiden, Melinda A. Beck, Christopher Hamilton-West, Stacey Schultz-Cherry

**Affiliations:** University of Chile, Santiago, Chile (N. Bravo-Vasquez, C. Hamilton-West);; St. Jude Children’s Research Hospital, Memphis, Tennessee, USA (E.A. Karlsson, P. Jimenez-Bluhm, V. Meliopoulos, B. Kaplan, S. Marvin, V. Cortez, P. Freiden, S. Schultz-Cherry);; University of North Carolina, Chapel Hill, North Carolina, USA (M.A. Beck)

**Keywords:** influenza, swine influenza virus, South America, backyard farm, transmission, zoonoses, influenza A, H1N1 pandemic strain, backyard production system, viruses, H1N2, influenza, Chile

## Abstract

Phylogenetic analysis of the influenza hemagglutinin gene (HA) has suggested that commercial pigs in Chile harbor unique human seasonal H1-like influenza viruses, but further information, including characterization of these viruses, was unavailable. We isolated influenza virus (H1N2) from a swine in a backyard production farm in Central Chile and demonstrated that the HA gene was identical to that in a previous report. Its HA and neuraminidase genes were most similar to human H1 and N2 viruses from the early 1990s and internal segments were similar to influenza A(H1N1)pdm09 virus. The virus replicated efficiently in vitro and in vivo and transmitted in ferrets by respiratory droplet. Antigenically, it was distinct from other swine viruses. Hemagglutination inhibition analysis suggested that antibody titers to the swine Chilean H1N2 virus were decreased in persons born after 1990. Further studies are needed to characterize the potential risk to humans, as well as the ecology of influenza in swine in South America.

In the past decade, variant swine-origin influenza A viruses (swIAVs) of subtypes H1N2 and H3N2 have been associated with human infections, particularly in persons with close contact to swine (*1*–*7*), although evidence of human-to-human transmission is minimal. The emergence and subsequent pandemic caused by the unique influenza A(H1N1)pdm09 virus (pH1N1) from swine in 2009 highlights the public health threat posed by swIAVs, especially in populations having little preexisting immunity (*8*). Therefore, it is imperative to understand the circulation of swIAVs on a global level.

While our knowledge of the global diversity and evolution of swIAVs has increased, less information has been available regarding swIAVs circulating in South America. Recently, subtypes H1N1, H1N2, and H3N2 were identified in commercial pigs in Chile (*9*). Phylogenetic analysis from 18 swIAVs sequenced from a 2012 cross-sectional sampling of 22 production companies in Chile (95% of their commercial swine) demonstrated extensive genetic diversity in the hemagglutinin (HA) segment (*9*). The human seasonal H1-like viruses were unique to swine in Chile and not closely related to other H1 swIAVs. These viruses represented 2 independent introductions from humans, with Chile H1 human I clade most closely related to human viruses from the late 1980s and Chile H1 human II clade most closely related to human viruses from the early 1990s (*9*). No further phylogenetic or virus characterization information was available.

In addition to commercial pig farming, Chile has an extensive backyard production system, a common form of animal production throughout the world. Backyard production farms (BPFs) are a particular public health concern given poor biosecurity and close interaction between humans and multiple animal species (*10*). During 2014, we conducted active backyard production system surveillance in Chile’s main swine production area (*11*) and isolated a subtype H1N2 swIAV virus (A/swine/Chile/YA026/2014; sw/Chile). We performed phylogenetic analyses and full genomic sequencing on this virus to determine its relationship with other known influenza isolates. We propagated it in primary human bronchial and swine respiratory epithelial cells, as well as C57Bl/6 mice, to determine its infectiousness. We also characterized its transmission using ferrets. Hemagglutination inhibition (HI) assays with human serum samples were done to assess the risk this virus poses for human infection, and serological analyses were conducted with pig serum samples from Chile to determine the prevalence of the virus and its risk for emergence. 

## Materials and Methods

### Study Area and Nasal Swab Sampling

Active surveillance was conducted as described (*11*) in 40 BPFs in Central Chile, representing 90% of the backyard production system. During this time, only 3 farms had swine (n = 3) available for sampling. We collected nasal samples using disposable sterile swabs, which were placed in 1 mL universal transport media. None of the animals were symptomatic.

### H1N2 Virus Isolation

We screened nasal swab samples by real-time reverse transcription PCR for matrix gene as described (*12*). PCR-positive samples were centrifuged at 8,000 rpm for 15 min and the clarified supernatant (200 µL) inoculated on MDCK cells in 24-well plates in duplicate and incubated at 37°C in 5% CO_2_ for 1 h. Following incubation, cells were washed then incubated with Eagle’s minimum essential medium (MEM; MediaTech, Manassas, VA, USA) supplemented with 2 mM glutamine, bovine serum albumin, and 1 µg/mL TPCK (L-1-tosylamide-2-phenylethyl chloromethyl ketone)–trypsin until cytopathic effect was observed. Then, cell supernatant was collected, centrifuged, and stored at −80°C as described (*12*). Only 1 virus was isolated: A/swine/Chile/YA026/2014 (H1N2) (sw/Chile).

### Cells

MDCK cells were cultured in MEM supplemented with 2 mM glutamine and 10% fetal bovine serum (Gemini BioProducts, West Sacramento, CA, USA) and grown at 37°C in 5% CO_2_. A549 cells were cultured in Dulbecco’s minimum essential medium (DMEM; Lonza, Walkersville, MD, USA) supplemented with 4.5 g/L glutamine and 10% fetal bovine serum and grown at 37°C in 5% CO_2_. Well-differentiated (transepithelial resistance >300 Ω × cm^2^), primary normal human bronchial epithelial (NHBE) cells were grown as previously described (*13*). In brief, cells were plated on collagen-coated membranes in bronchial epithelial basal media (Lonza) supplemented with SingleQuots additives (Lonza) and media changed every 48 h. When cells became 100% confluent, basal media was replaced with DMEM:F12 (Lonza) supplemented with SingleQuots, and apical media was removed. Every 48–72 h, the apical surface was washed with 0.9% sodium chloride solution (Sigma, St. Louis, MO, USA). Cells were incubated at the air-liquid interface at 37°C in 5% CO_2_. Primary swine nasal epithelial cells (sNECs) were prepared as described (*14*). In brief, nasal swab specimens were collected, placed in 50 mL conical tubes, and incubated in phosphate-buffered saline (PBS) at 4°C for 10–15 min before centrifuging for 10 min at 300 × *g* at 4°C. The pellet was resuspended in supplemented DMEM:F12 and incubated on collagen-coated plates at 37°C in 5% CO_2._ Cells were plated onto transwells and, when 100% confluent, moved to an air–liquid interface to differentiate for 1–3 wk. sNECs were moved to 33°C 1 d before infection.

### Virus Sequencing

Sanger sequencing of viral stocks was performed by the St. Jude Hartwell Center (*12*), and deep amplicon sequencing and assembly were conducted as described previously (*15*). Sequences can be obtained in GenBank (accession nos. CY207245–CY207252).

### Viral Propagation

A/swine/Chile/YA026/2014 (H1N2, sw/Chile), A/Memphis/3/1987 (H1N1, Mem/87), A/swine/Iowa/13-1015/2010 (H1N2, sw/IA), A/swine/Indiana/26-0818/2011 (H1N2, sw/IN), A/swine/North Carolina/18161/2002 (H1N1, sw/NC), A/swine/Italy/1310-2/1995 (H1N1, sw/IT), and A/California/04/2009 (pH1N1, CA/09) viruses were propagated in the allantoic cavity of 10-day-old, pathogen-free, embryonic chicken eggs at 37°C and viral titers determined by 50% tissue culture infectious dose (TCID_50_) analysis as previously described (*16*,*17*). These viruses were used as comparators for all studies except the ferret experiments.

### Phylogenetic Analysis

We performed sequence assembly and editing by using BioEdit version 7.2.5 (*18*) and sequence alignment with MUSCLE version 3.8.3 (*19*). Reference sequences were obtained from the Influenza Virus Resource at NCBI (*20*). Phylogenic relationships for each gene was inferred by maximum likelihood, incorporating a general time-reversible model of nucleotide substitution with a gamma-distributed rate variation among sites by using RAxML version 8.0 (*21*). A bootstrap resampling process of 200 replicates was implemented to provide statistical robustness to each node.

### Swine and Human Serum Samples

During 2013–2015, swine serum samples (n = 266, 1–24 animals/BP) were collected from 80 BPFs in Central Chile, including the Valparaiso, Metropolitan, and Libertador General Bernardo O’Higgins regions. During 2009–2015, human serum samples were collected as part of an ongoing prospective observational study at the University of North Carolina Family Medicine Center (Chapel Hill, NC, USA). We chose a subset of these samples, collected 28–32 d after seasonal influenza vaccination; grouped them according to birth decade (1920–1929, 1930–1939, 1940–1949, 1950–1959, 1960–1969, 1970–1979, 1980–1989, and 1990–1999; n = 7–22/age group); and balanced for sex and race whenever possible (*22*).

### Hemagglutination Inhibition Assay

Ferret antiserum was generated against sw/Chile, Mem/87, sw/IA, CA/09, sw/IN, sw/NC, and sw/IT viruses. HI assay was conducted as previously described (*23*). In brief, antiserum was treated with receptor-destroying enzyme (Denka Seiken, Tokyo, Japan), inactivated, and diluted 1:10. Serial dilutions of receptor-destroying enzyme–treated serum were incubated in duplicate with each virus for 15 min at room temperature, followed by 30 min incubation at 4°C with 0.5% turkey erythrocytes. HI titer was defined as the reciprocal dilution of the last well that reacted.

### In Vitro Replication

MDCK cells were infected with a multiplicity of infection (MOI) of 0.01 for 1 h at 37°C. Cells were washed 3 times to remove unbound virus, and infected cells were cultured in MEM containing 0.075% bovine serum albumin and 1 μg/mL TPCK-treated trypsin. Aliquots of culture supernatants were collected at 6 h postinfection (hpi), 24 hpi, 48 hpi, and 72 hpi and immediately stored at −80°C. For infection of NHBE cells and sNECs, the apical surface was washed twice and incubated with serum-free DMEM containing virus for 2 h at 37°C, after which both apical and basal media were removed and fresh growth medium was added to the basal chamber as described (*24*). At 6 hpi, 24 hpi, 48 hpi, 72 hpi, and 96 hpi, DMEM was added to the apical surface, and cells were incubated for 30 min at 37°C (for NHBE) or 33°C (for sNEC). Apical media was collected and stored at −80°C. Viral titers were determined by TCID_50_ on MDCK cells followed by Reed-Munch analysis (*25*).

### Animal Infections

Six- to 8-week-old female BALB/c mice (Jackson Laboratory, Bar Harbor, ME, USA) (n = 11 mice/group) were lightly anesthetized with isofluorane and intranasally inoculated with PBS or 10^5^ TCID_50_ units of virus in 25 μL PBS. Mice were monitored daily for signs of infection and weighed every 48 hpi (*26*). At 3 d postinfection (dpi) and 6 dpi, 3 control and 3 infected mice were humanely killed; lungs were harvested and homogenized in 1 mL PBS and nasal washes were collected as previously described (*27*). In brief, an incision was made in the trachea and 500 µL of PBS was flushed through the upper respiratory tract and collected. Viral titers were determined by TCID_50_ (*16*,*24*). Data are representative of 2 separate experiments. For transmission studies, 9- to 15-week-old male ferrets (n = 2, Triple F Farms, Sayre, PA, USA) were intranasally inoculated with 10^6^ TCID_50_ units in 1 mL PBS. Twenty-four hours later, naive ferrets (n = 2 mice/group) were either placed in direct contact with infected ferrets or housed in separate cages (respiratory contact) separated by ≈5 inches. Nasal washes were collected every 2 dpi for viral titration and serum samples collected at 21 dpi for HI analysis as described (*28*).

### Ethics Statement

All sampling was approved by the ethics and biosecurity committee of Faculty of Veterinary Science, University of Chile, and collected by trained veterinarians according to Food and Agriculture Organization guidelines (http://www.fao.org/3/a-ak738e.pdf). Animal experiments were approved by the St. Jude Children’s Research Hospital institutional biosafety committee and animal care and use committee and were in compliance with the Guide for the Care and Use of Laboratory Animals. These guidelines were established by the Institute of Laboratory Animal Resources and approved by the Governing Board of the US National Research Council. All human procedures were approved by the Biomedical Institutional Review Board at the University of North Carolina.

### Statistical Analysis

We performed statistical analyses using JMP statistical software (SAS Institute, Cary, NC, USA) and GraphPad Prism software (GraphPad Software Inc., La Jolla, CA, USA). The Kruskal-Wallis test (α = 0.05) was used to analyze nonparametric data, while normally distributed data were analyzed by 2-way analysis of variance with virus strain and time postinfection as main effects. The Student *t*-test was used for posthoc comparisons between the viruses, and Tukey's honest significant difference was used for posthoc comparisons among the times postinfection. Differences were considered significant at p<0.05.

## Results

### Phylogenetic Similarity of Chilean H1N2 and Human Seasonal Viruses

**Figure 1:**
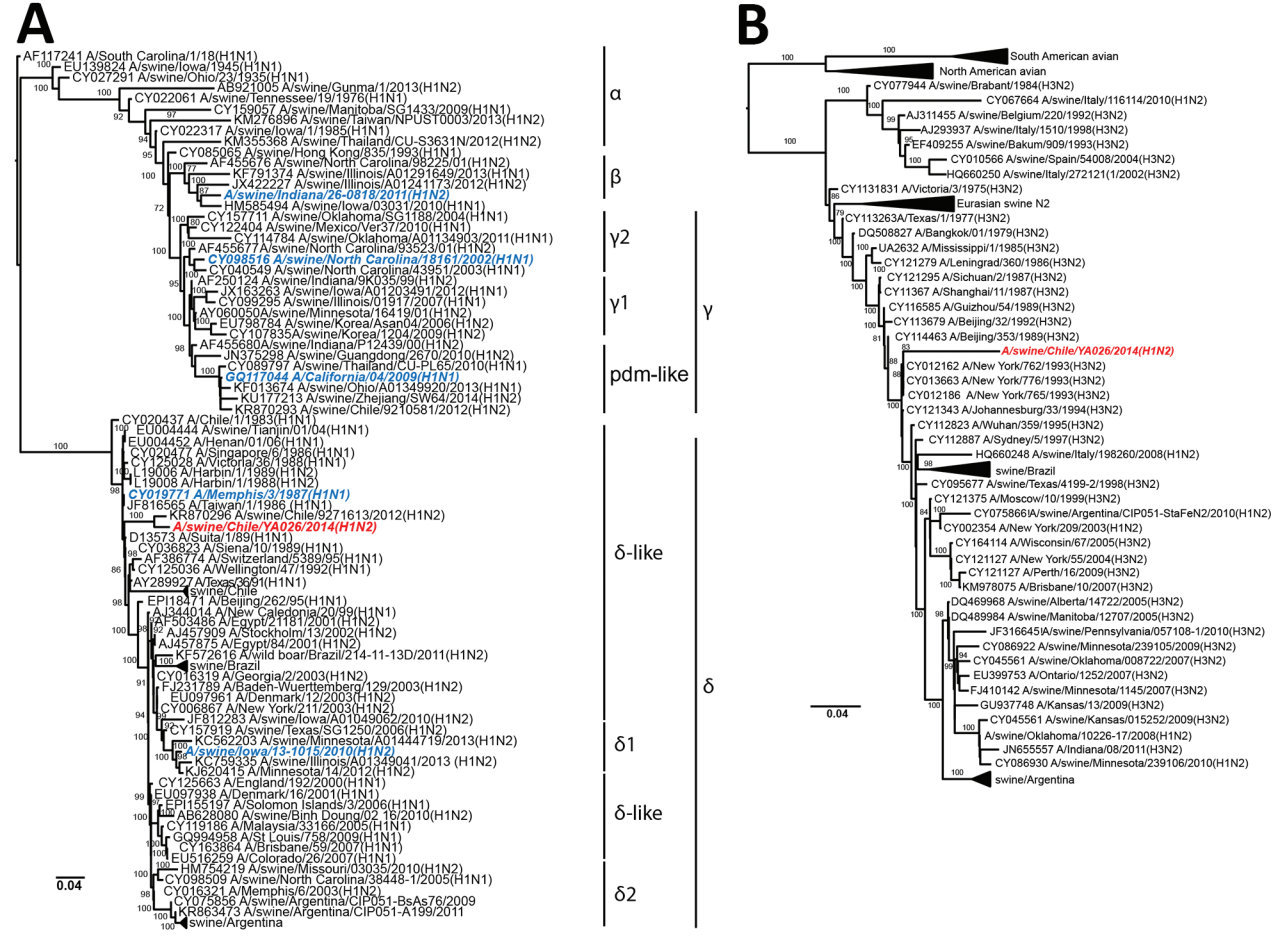
Phylogenetic trees showing comparison of swine influenza virus (H1N2) from Chile (red) and reference viruses. We performed phylogenetic analyses on complete hemagglutinin (A) and neuraminidase (B) genome sequences using RAxML with 200-bootstrap replicates (*21*). Blue indicates control H1 viruses. Scale bars indicate number of substitutions per site.

Phylogenetic analysis demonstrated that sw/Chile H1N2 belongs to the δ-like H1 subgroup (Figure 1, panel A) and is phylogenetically similar to the swIAVs from Chile described by Nelson et al. (*9*). The HAs of these swIAVs are closely related to the HAs of human seasonal H1 viruses from the late 1980s and early 1990s (closest relative A/Suita/1/89, 94% nucleotide identity), although they are separated by long branch lengths and represent over 15 years of evolution (*9*). Unfortunately, only the HA segments from the 2012 Chilean swIAVs were available in public databases, so we were unable to compare full genomes. 

**Figure 2:**
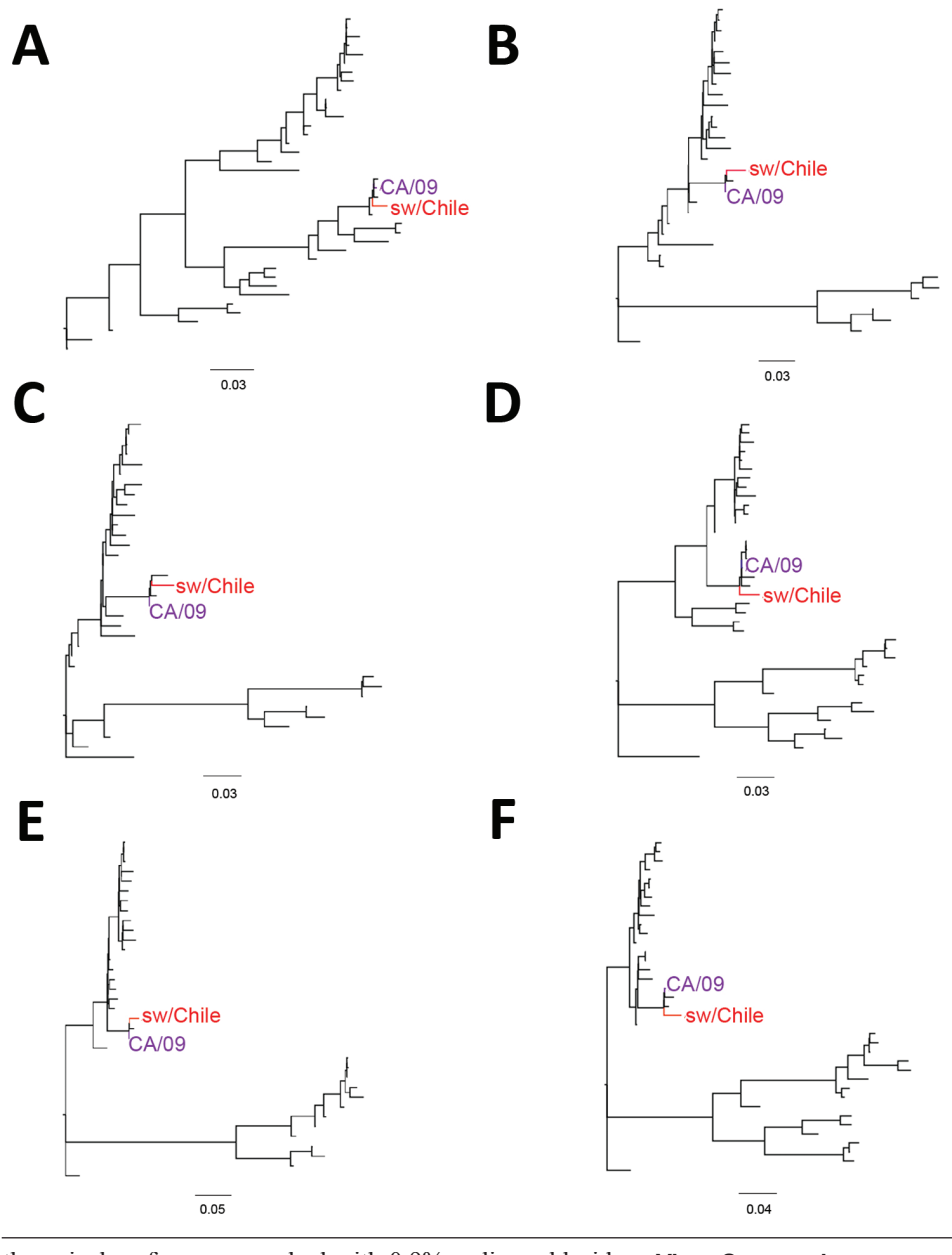
Phylogenetic trees comparing the internal genes of swine influenza virus (H1N2) from Chile (red) and reference viruses. We performed phylogenetic analysis for the matrix (A), nucleoprotein (B), nonstructural (C), polymerase acid (D), polymerase basic (PB) 1 (E), and PB2 (F) gene segments by using RAxML with 200-bootstraps replicates (*21*). Purple indicates location of control influenza A(H1N1)pdm09 CA/09 virus. Scale bars indicate number of substitutions per site. Detailed phylogenetic trees for these genes are provided in Technical Appendix Figures 1–6.

The sw/Chile neuraminidase (NA) segment arose from a reassortment between human seasonal H1N1 and H3N2 viruses (Figure 1, panel B). However, long branch length indicates extensive genetic divergence from human N2 sequences, with the closest sequence being an early 1990s North American H3N2 virus (A/New York/762/1993, 93% nucleotide identity). This difference can be indicative of possible temporal and species-specific adaptations, leading to genetic and antigenic differences with contemporary human N2 viruses. The sw/Chile virus internal genes are pH1N1 in origin, consistent with the high reassortment rate between pH1N1 and swine viruses (Figure 2; Technical Appendix Figures 1–6) (*9*,*29*). Overall, while sw/Chile does appear to originally be of human origin, detection of these viruses in multiple BPFs suggests these viruses might have become established in the swine population in Chile.

### Antigenicity and Seroprevalence of Chilean H1N2 Virus

**Figure 3:**
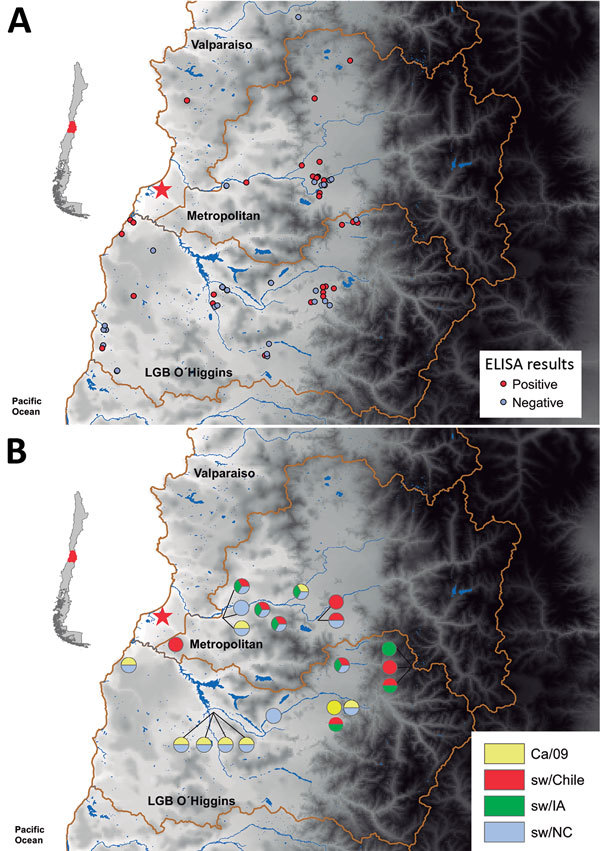
Testing for influenza in swine from backyard production farms (BPFs) in Central Chile. Swine serum samples collected from BPFs were analyzed for antibodies against influenza by nucleoprotein (NP)–specific ELISA, and hemagglutination inhibition analysis of serum samples from NP ELISA–positive farms was conducted to determine subtype. A) Results of influenza testing by BPF location. Red circles indicate location of positive farms, and gray circles indicate location of negative farms. B) Pie charts showing percentage of swine at each sampling site seropositive for each virus type: sw/Chile (red), CA/09 (yellow), sw/IA (green), and sw/NC (blue). Red star indicates location of sw/Chile H1N2 virus isolate. Insets indicate region of Central Chile covered by this study (red shading).

HI assays with a panel of ferret antiserum against classical swIAV (sw/IN and sw/NC from β and γ subgroups, respectively); pandemic CA/09 virus; sw/Iowa (from δ subgroup); and human seasonal H1 virus Mem/87 showed that sw/Chile H1N2 virus was antigenically distinct from other H1 viruses (Table). To determine seroprevalence in swine within the surveillance area, serum samples (n = 266, 1–24 animals/BPF) were collected from swine on 80 BPFs in Central Chile in 2013–2015. Forty-eight (60%) BPFs were positive by nucleoprotein-specific ELISA, and the prevalence of positive pigs (86/266, 32.3%) on each BPF ranged from 14.2% to 100% animals (Figure 3, panel A). HI assays performed with a panel of influenza viruses on the ELISA-positive samples demonstrated that 14/48 (29.1%) BPFs and 22/86 pigs were positive for >1 of the test viruses. Antibodies against sw/Chile were identified in BPFs located in 3 regions of Central Chile (Figure 3, panel B). Of concern, 11/14 (78.6%) of the positive BPFs, representing 15/22 (68.2%) of the positive samples, were positive for multiple influenza strains, suggesting that different influenza viruses are co-circulating in backyard swine in Central Chile (Figure 3, panel B). Not all nucleoprotein ELISA–positive samples could be subtyped.

**Table:** Antigenic characteristics of swine influenza virus (H1N2) from Chile and control viruses*

Virus	Subtype	Major clade	Subclade	Ferret antiserum results					
				Mem/87	sw/Chile	sw/IA	sw/NC	sw/IT	CA/09
Mem/87	hsH1N1	North America	δ	640	<	<	<	<	<
sw/Chile	swH1N2	North America	δ	<	640	<	<	<	<
sw/Iowa	swH1N2	North America	δ1	<	<	320	<	<	<
sw/NC	swH1N1	North America	γ2	<	<	<	80	<	80
sw/It	swH1N1	Eurasian	-	<	<	<	<	320	40
CA/09	pH1N1	North America	pdm-like	<	<	<	<	<	320
sw/In	swH1N2	North America	β	<	<	<	320	160	40

*< refers to titers being below the minimum detectable hemagglutinin inhibition titer of 1:10. CA/09, A/California/04/2009; hs, human seasonal; MEM/87, A/Memphis/3/1987; pdm, pandemic; pH1N1, influenza A(H1N1)pdm09 virus; sw, swine; sw/Chile, A/swine/Chile/YA026/2014; sw/IA, A/swine/Iowa/13-1015/2010; sw/IN, A/swine/Indiana/26-0818/2011; sw/IT, A/swine/Italy/1310-2/1995; sw/NC, A/swine/North Carolina/18161/2002.

### Chile H1N2 Virus in Primary Swine and Human Cells 

**Figure 4:**
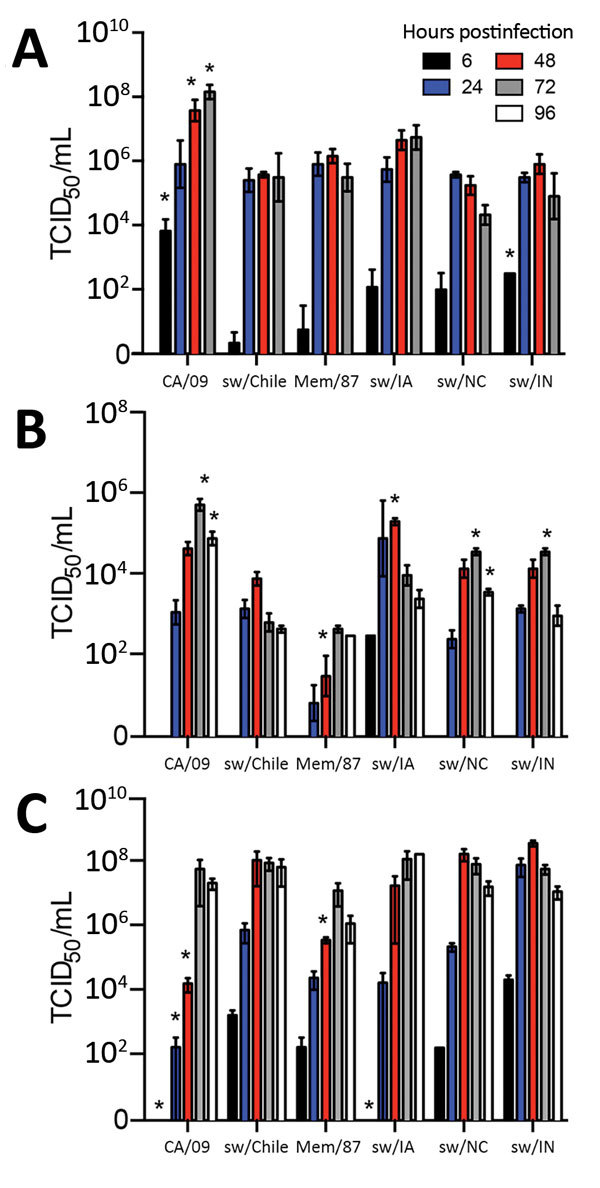
Replication of influenza viruses in vitro. MDCK cells (A), primary normal human bronchial epithelial cells (B), or primary swine nasal epithelial cells (C) were grown in an air-liquid interface and infected with the indicated viruses at a multiplicity of infection of 0.01. Cell culture supernatants were collected at 6 hours postinfection (hpi), 24 hpi, 48 hpi, and 72 hpi, for MDCK cells and 6 hpi, 24 hpi, 48 hpi, 72 hpi, and 96 hpi for primary normal human bronchial epithelial cells and primary swine nasal epithelial cells, and viral titers were determined by 50% tissue culture infectious dose (TCID_50_) analysis. Data are presented as mean titer of 3 replicates ± SEM. *p<0.05 versus sw/Chile virus.

Swine possess both mammalian (α-2,6) and avian (α-2,3) influenza receptors (*30*,*31*). Receptor binding assays (*23*) revealed that all viruses used in this study had greater binding affinity for mammalian-type (α-2,6–linked sialic acid) receptors (Technical Appendix Figure 7). These viruses also replicated effectively in MDCK cells; differentiated, primary NHBE cells; and sNECs cultured at the air-liquid interface. Cells were infected at MOI 0.01 and viral titers determined at the indicated times by TCID_50_ analysis on MDCK cells (*23*). All viruses replicated to similar titers in MDCK cells (Figure 4, panel A) and sNECs (Figure 4, panel C), except for an increased replication of CA/09 virus in MDCK cells. However, significant differences in replication were observed in primary NHBE cells. The human seasonal Mem/87 and sw/Chile viruses replicated to lower titers than the other swine viruses, suggesting reduced fitness in human cells (Figure 4, panel B). Overall, these studies demonstrate that sw/Chile can productively replicate in human and swine cells.

### Chile H1N2 Virus in Mice

**Figure 5:**
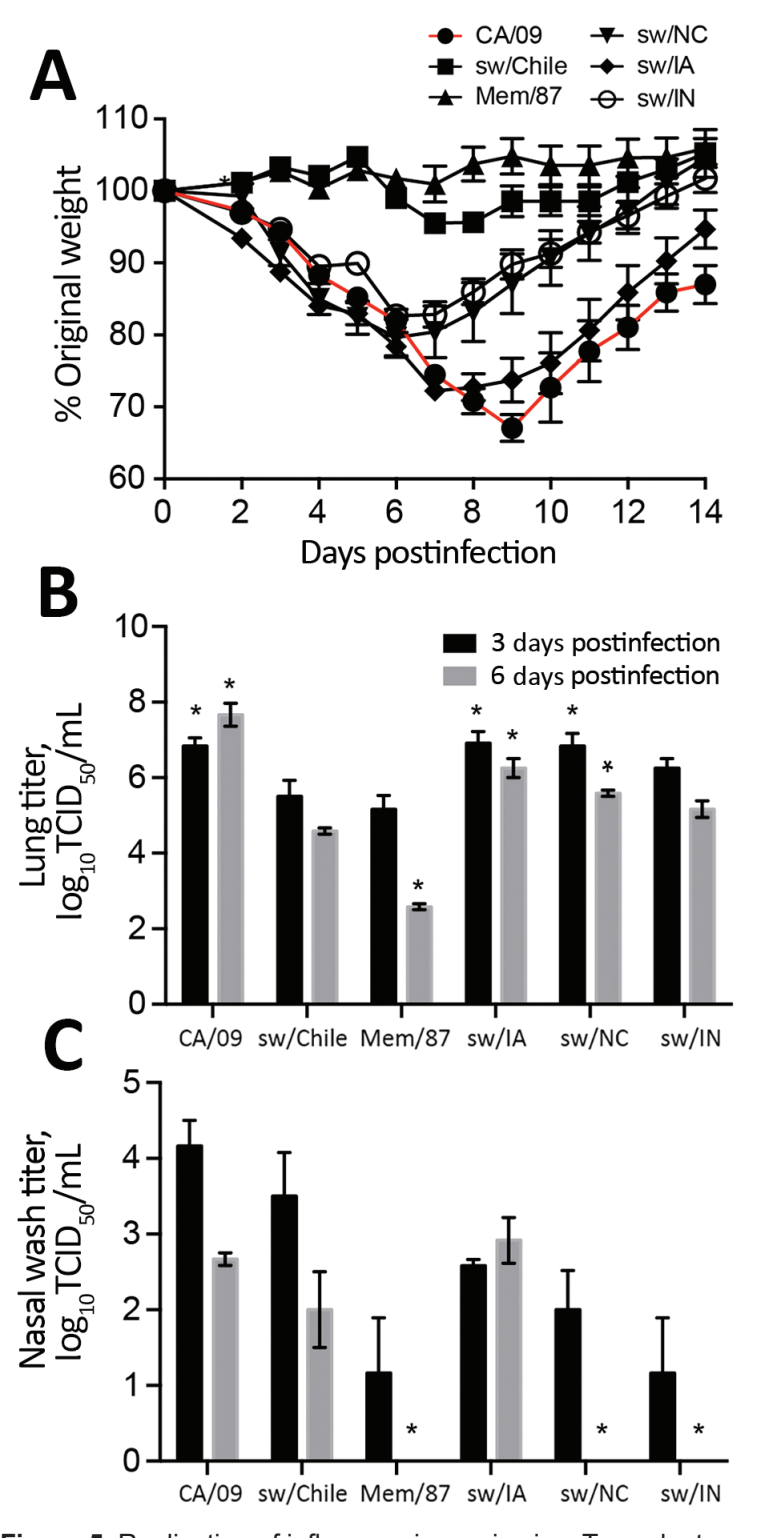
Replication of influenza viruses in vivo. To evaluate pathogenicity in mice, 6- to 8-week-old BALB/c mice (n = 11 mice/group/experiment) were infected with 10^5^ 50% tissue culture infectious dose (TCID_50_) units of the indicated viruses and weight loss was monitored for 14 days postinfection (dpi) (A). At 3 dpi and 6 dpi, lungs (B) and nasal washes (C) were collected from 3 mice/group and viral titers were determined by TCID_50_ analysis. Data are presented as mean ± SEM. *p<0.05 versus sw/Chile virus.

Recently identified swine H1N2 viruses from China replicated efficiently in mice and pigs (*32*); however, no data were available on viruses from South America. Thus, we intranasally inoculated Balb/c mice with 10^5^ TCID_50_ units of each virus and monitored weight loss for 14 dpi. Viral titers were measured 3 dpi and 6 dpi (Figure 5). CA/09 virus caused considerable weight loss, as did sw/IA (H1N2, δ1 subclade), which was similar to CA/09, sw/IN (H1N2, β subclade), and sw/NC (H1N1, γ2 subclade) viruses. Increased weight loss was associated with efficient replication in lungs (Figure 5, panel B). In contrast, the Mem/87 and sw/Chile viruses caused minimal illness and had lower replication in lungs (Figure 5, panels A and B). The sw/Chile virus replicated the most effectively in the upper respiratory tract compared with the other swine viruses, which reached titers similar to that of CA/09 virus (Figure 5, panel C). These data show that while sw/Chile virus can replicate in mice, it causes minimal illness. The genetic basis for differences in pathogenicity and tropism between the swine strains warrants further investigation.

### Chile H1N2 Virus in Respiratory Droplets

**Figure 6:**
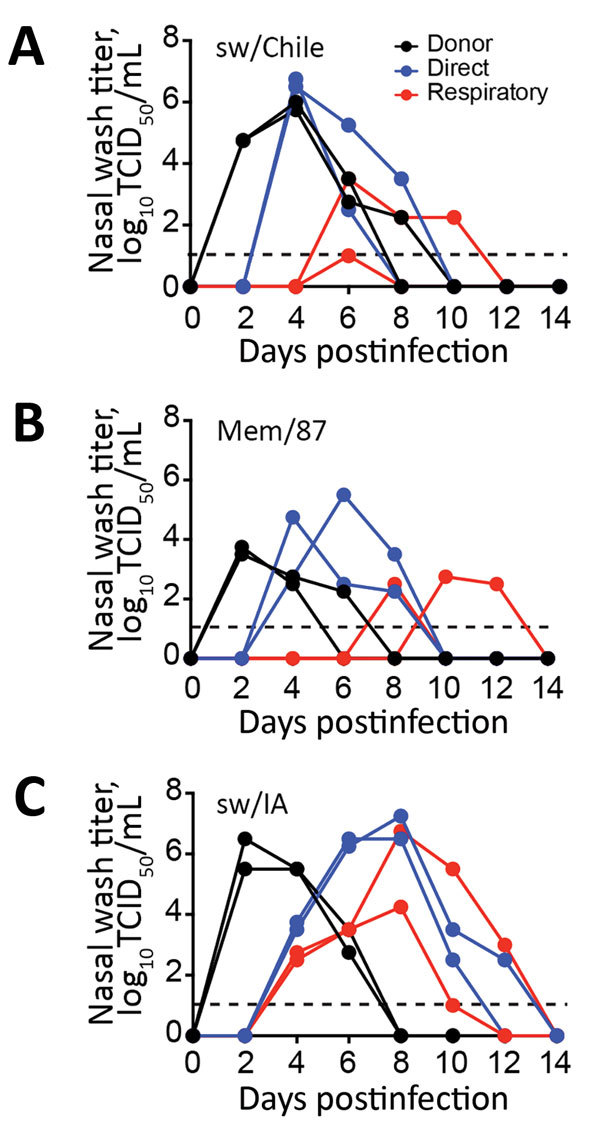
Evaluation of H1 virus transmission in ferrets. Donor ferrets (black lines; n = 2 ferrets/group) were inoculated with 10^6^ 50% tissue culture infectious dose (TCID_50_) units of H1 influenza virus sw/Chile (A), Mem/87 (B), or sw/IA (C) viruses. Naive ferrets (n = 2) were either placed in the same cage with the infected group (direct contact, blue lines) or housed in separate cages (respiratory transmission, red lines) and nasal washes were collected on the indicated day postinfection for virus quantification by TCID_50_ analysis. Lines represent individual animals. Dashed line represents limit of detection of the assay.

Eurasian swine H1N2 viruses transmit in ferrets by direct contact and, in some cases, by respiratory droplet (*33*,*34*). To test the transmissibility of sw/Chile virus, we intranasally inoculated ferrets with 10^6^ TCID_50_ units of sw/Chile, Mem/87, or sw/IA viruses; at 1 dpi, naive ferrets were either placed in direct contact or housed in cages adjacent to the donor ferrets to monitor respiratory droplet transmission. Nasal washes were collected every 2 dpi to assess viral shed, and animals were monitored through 21 dpi. All of the donor animals shed virus, and all viruses transmitted through direct contact and respiratory droplet with some differences (Figure 6). Overall, the swine viruses replicated to a higher titer than the Mem/87 virus did in the donor and direct contact animals; swine viruses also transmitted faster by respiratory droplet. Respiratory contacts of sw/Chile virus–inoculated animals were shedding virus by 6 dpi, whereas Mem/87 virus–inoculated animals only showed detectable nasal washes after 8 dpi and 10 dpi. The sw/IA virus appeared to transmit faster and replicate to higher titer than did the sw/Chile and Mem/87 viruses (Figure 6). All animals seroconverted at 21 dpi. These studies highlight that both swine H1 δ viruses transmitted effectively in ferrets.

### Decreased HI Antibody Titer in Persons Born after 1990 

**Figure 7:**
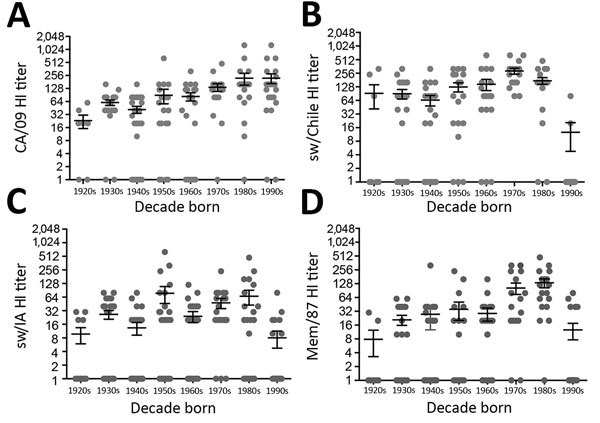
Evaluation of serologic responses of humans to H1 viruses by age. Hemagglutination inhibition (HI) studies were performed for CA/09 (A), sw/Chile (B), sw/IA (C), and Mem/87 (D) viruses by using human serum samples (n = 137) collected as part of an ongoing prospective observational study carried out at the University of North Carolina Family Medicine Center (Chapel Hill, NC, USA) in 2009–2015. A subset of the available samples were chosen from persons whose serum samples were collected 28–32 days after seasonal influenza vaccination. Samples were grouped by decade of birth (1920–1929, 1930–1939, 1940–1949, 1950–1959, 1960–1969, 1970–1979, 1980–1989, and 1990–1999). Groups consisted of samples from 7–22 persons and were balanced for sex and age whenever possible. Gray dots represent inverse HI titers of individual persons and bars represent mean HI titer ± SEM per decade born.

Because the HA and NA of sw/Chile were derived from human seasonal strains originating around 1990, we hypothesized that persons born after 1990 would have decreased HI antibody titers. To test this, we conducted HI analyses against CA/09, Mem/87, sw/IA, and sw/Chile viruses on serum samples from 137 persons in North America collected in 2010–2015; samples were grouped by decade of birth (n = 7–22 samples/group). Most persons born before 1990 had titers against all 4 viruses (Figure 7). However, few persons born after 1990 had titers against sw/Chile (9%; Figure 7, panel B); sw/IA (31.8%; Figure 7, panel C); or Mem/87 (27.2%; Figure 7, panel D) viruses. Persons with titers ranging from 1:10 to 1:160 were all born in 1990 or 1991. In contrast, most (91.7%) persons born after 1990 had titers against pH1N1 virus, ranging from 1:320 to 1:10,240 (Figure 7, panel A).

## Discussion

Our data show that the swine H1N2 virus from Chile is antigenically distinct from other H1 viruses, replicates efficiently in mammalian cells, can transmit by respiratory droplet, and might pose a risk to immunologically naive persons born after 1990. Our study furthers the data recently published on prevalence and HA phylogeny of Chile swIAVs (*9*). We show that these viruses are circulating outside of commercial swine herds in BPFs, where animal–human interaction is high.

Since emergence of pH1N1 in 2009, reverse transmission of the virus from humans to pigs has been documented worldwide, resulting in several reassortments with classical swine and seasonal human viruses (*1*,*2*,*4*,*5*,*9*,*12*,*35*). As of July 2016, variant (v) influenza viruses have caused over 380 human infections in the United States alone. While most of these infections have been H3N2v viruses, 8 have been H1N2v viruses. These infections are particularly concerning because most infections occurred in children (persons <18 years of age) who had direct or indirect exposure to swine (*2*). Census estimates indicate that 50% of BPFs in Chile have >1 person born after 1990, and 35% have >1 person born after 2000 (C. Hamilton-West, unpub. data). Our data indicate that younger age groups have reduced HI titers to sw/Chile virus; however, studies with serum samples obtained from throughout the population of Chile are warranted to better determine the risk these viruses pose to swine herds.

This study is subject to several limitations. First, these studies were limited to 1 virus isolate. A panel of swine viruses from Chile isolated from different years and regions warrant study, especially if genetic differences are found among viruses. Second, these results represent a limited number of the backyard production system within Chile. Enhanced swine surveillance throughout Chile and South America is needed. Third, studies at the animal–human interface are required to decipher the risk circulating swIAVs pose to humans. We were limited to serum samples from persons living in North America, and further work should be done on South America populations, especially with those exposed to swine. Antigenic cartography on serum samples from persons involved in backyard farming would provide invaluable new information on transmission of swine viruses to humans. Finally, further work is needed to improve understanding of swIAV genes involved in transmission in the ferret model.

Despite no evidence of sustained human-to-human transmission, increased risk for human infection with H1N2v viruses calls for further study and enhanced monitoring. Risk for possible emergence of zoonotic strains has been demonstrated worldwide. H1N2 viruses isolated from pigs in China replicated efficiently in mice and pigs (*32*,*36*,*37*), while viruses from South Korea and Europe transmitted to and even caused death in ferrets (*33*,*34*,*38*). Future work will focus on how these reassortants arose in Chile BPFs and the role of specific genetic differences in pathogenicity and transmissibility. In addition, further studies for seroprevalence in Chile BPF workers and antigenic cartography with the sw/Chile virus are necessary. Studies at the animal–human interface are needed to determine seroprevalence, identify broadly protective antibodies, and characterize T-cell responses, and active surveillance is needed to uncover infection rates to better determine the risk for zoonotic transmission of swine-origin H1N2 to human health. In summary, our findings highlight the need for continued, vigilant influenza virus surveillance in Chile and throughout South America.

Technical Appendix Phylogenetic trees of the internal genes of swine influenza virus (H1N2) from Chile, H1 virus α-2,6–linked sialic acid binding affinities, and H1 virus disease induction assessed by weight and temperature changes in infected ferrets.

## References

[1] Komadina N, McVernon J, Hall R, Leder K. A historical perspective of influenza A(H1N2) virus. Emerg Infect Dis. 2014;20:6–12. 10.3201/eid2001.121848 24377419 PMC3884707

[2] Centers for Disease Control and Prevention. Reported infections with variant influenza viruses in the United States since 2005. 2016 Sep 12 [cited 2016 Oct 10]. http://www.cdc.gov/flu/swineflu/variant-cases-us.htm

[3] Herriman R. Swine flu, H1N2v virus infections reported in Wisconsin, Minnesota. 2016 Jul 1 [cited 2016 Oct 10]. http://outbreaknewstoday.com/swine-flu-h1n2v-virus-infections-reported-in-wisconsin-minnesota-22255/

[4] Centers for Disease Control and Prevention. Influenza A (H3N2) variant virus [cited 2016 Oct 6]. http://www.cdc.gov/flu/swineflu/h3n2v-cases.htm

[5] Centers for Disease Control and Prevention. H1N2 variant virus detected in Minnesota. 2012 Sep 7 [cited 2016 Oct 6]. http://www.cdc.gov/flu/spotlights/h1n2v-cases-mn.htm

[6] Trombetta C, Piccirella S, Perini D, Kistner O, Montomoli E. Emerging influenza strains in the last two decades: a threat of a new pandemic? Vaccines (Basel). 2015;3:172–85. 10.3390/vaccines3010172 26344952 PMC4494236

[7] Choi MJ, Morin CA, Scheftel J, Vetter SM, Smith K, Lynfield R; Variant Influenza Investigation Team. Variant influenza associated with live animal markets, Minnesota. Zoonoses Public Health. 2015;62:326–30. 10.1111/zph.12139 24931441

[8] Dawood FS, Jain S, Finelli L, Shaw MW, Lindstrom S, Garten RJ, et al.; Novel Swine-Origin Influenza A (H1N1) Virus Investigation Team. Emergence of a novel swine-origin influenza A (H1N1) virus in humans. N Engl J Med. 2009;360:2605–15. 10.1056/NEJMoa0903810 19423869

[9] Nelson M, Culhane MR, Rovira A, Torremorell M, Guerrero P, Norambuena J. Novel human-like influenza A viruses circulate in swine in Mexico and Chile. PLoS Curr. 2015;7:7. 26345598 10.1371/currents.outbreaks.c8b3207c9bad98474eca3013fa933ca6 PMC4551470

[10] Hamilton-West C, Rojas H, Pinto J, Orozco J, Hervé-Claude LP, Urcelay S. Characterization of backyard poultry production systems and disease risk in the central zone of Chile. Res Vet Sci. 2012;93:121–4. 10.1016/j.rvsc.2011.06.015 21752410

[11] Bravo-Vasquez N, Di Pillo F, Lazo A, Jiménez-Bluhm P, Schultz-Cherry S, Hamilton-West C. Presence of influenza viruses in backyard poultry and swine in El Yali wetland, Chile. Prev Vet Med. 2016;134:211–5. 10.1016/j.prevetmed.2016.10.004 27726887

[12] Karlsson EA, Ciuoderis K, Freiden PJ, Seufzer B, Jones JC, Johnson J, et al. Prevalence and characterization of influenza viruses in diverse species in Los Llanos, Colombia. Emerg Microbes Infect. 2013;2:e20. 10.1038/emi.2013.20 26038461 PMC3636595

[13] Fulcher ML, Randell SH. Human nasal and tracheo-bronchial respiratory epithelial cell culture. In: Randell HS, Fulcher LM, editors. Epithelial cell culture protocols, 2nd ed. Totowa (NJ): Humana Press; 2013. p. 109–21. 10.1007/978-1-62703-125-7_8 23097104

[14] Dash P, Barnett PV, Denyer MS, Jackson T, Stirling CMA, Hawes PC, et al. Foot-and-mouth disease virus replicates only transiently in well-differentiated porcine nasal epithelial cells. J Virol. 2010;84:9149–60. 10.1128/JVI.00642-10 20592089 PMC2937594

[15] Kaplan BS, Russier M, Jeevan T, Marathe B, Govorkova EA, Russell CJ, et al. Novel highly pathogenic avian A(H5N2) and A(H5N8) influenza viruses of clade 2.3.4.4 from North America have limited capacity for replication and transmission in mammals. mSphere. 2016;1:piie00003-16. 10.1128/mSphere.00003-16 27303732 PMC4894690

[16] Jones JC, Turpin EA, Bultmann H, Brandt CR, Schultz-Cherry S. Inhibition of influenza virus infection by a novel antiviral peptide that targets viral attachment to cells. J Virol. 2006;80:11960–7. 10.1128/JVI.01678-06 17005658 PMC1676284

[17] Carlson CM, Turpin EA, Moser LA, O’Brien KB, Cline TD, Jones JC, et al. Transforming growth factor-β: activation by neuraminidase and role in highly pathogenic H5N1 influenza pathogenesis. PLoS Pathog. 2010;6:e1001136. 10.1371/journal.ppat.1001136 20949074 PMC2951376

[18] Hall TA. BioEdit: a user-friendly biological sequence alignment editor and analysis program for Windows 95/98/NT. Nucleic Acids Symp Ser. 1999;41:95–8.

[19] Edgar RC. MUSCLE: multiple sequence alignment with high accuracy and high throughput. Nucleic Acids Res. 2004;32:1792–7. 10.1093/nar/gkh340 15034147 PMC390337

[20] Bao Y, Bolotov P, Dernovoy D, Kiryutin B, Zaslavsky L, Tatusova T, et al. The influenza virus resource at the National Center for Biotechnology Information. J Virol. 2008;82:596–601. 10.1128/JVI.02005-07 17942553 PMC2224563

[21] Stamatakis A. RAxML version 8: a tool for phylogenetic analysis and post-analysis of large phylogenies. Bioinformatics. 2014;30:1312–3. 10.1093/bioinformatics/btu033 24451623 PMC3998144

[22] Sheridan PA, Paich HA, Handy J, Karlsson EA, Hudgens MG, Sammon AB, et al. Obesity is associated with impaired immune response to influenza vaccination in humans. Int J Obes. 2012;36:1072–7. 10.1038/ijo.2011.208 22024641 PMC3270113

[23] Karlsson EA, Ip HS, Hall JS, Yoon SW, Johnson J, Beck MA, et al. Respiratory transmission of an avian H3N8 influenza virus isolated from a harbour seal. Nat Commun. 2014;5:4791. 10.1038/ncomms5791 25183346 PMC4801029

[24] Cline TD, Karlsson EA, Freiden P, Seufzer BJ, Rehg JE, Webby RJ, et al. Increased pathogenicity of a reassortant 2009 pandemic H1N1 influenza virus containing an H5N1 hemagglutinin. J Virol. 2011;85:12262–70. 10.1128/JVI.05582-11 21917948 PMC3209346

[25] Reed LJ, Muench H. A simple methods of estimating fifty percent endpoints. Am J Epidemiol. 1938;27:493–7.

[26] Morton DB. A systematic approach for establishing humane endpoints. ILAR J. 2000;41:80–6. 10.1093/ilar.41.2.80 11406701

[27] Zhou B, Meliopoulos VA, Wang W, Lin X, Stucker KM, Halpin RA, et al. Reversion of cold-adapted live attenuated influenza vaccine into a pathogenic virus. J Virol. 2016;90:8454–63. 10.1128/JVI.00163-16 27440882 PMC5021423

[28] Barman S, Krylov PS, Fabrizio TP, Franks J, Turner JC, Seiler P, et al. Pathogenicity and transmissibility of North American triple reassortant swine influenza A viruses in ferrets. PLoS Pathog. 2012;8:e1002791. 10.1371/journal.ppat.1002791 22829764 PMC3400563

[29] Nelson MI, Wentworth DE, Culhane MR, Vincent AL, Viboud C, LaPointe MP, et al. Introductions and evolution of human-origin seasonal influenza a viruses in multinational swine populations. J Virol. 2014;88:10110–9. 10.1128/JVI.01080-14 24965467 PMC4136342

[30] Suzuki Y, Ito T, Suzuki T, Holland RE Jr, Chambers TM, Kiso M, et al. Sialic acid species as a determinant of the host range of influenza A viruses. J Virol. 2000;74:11825–31. 10.1128/JVI.74.24.11825-11831.2000 11090182 PMC112465

[31] Ito T, Couceiro JNSS, Kelm S, Baum LG, Krauss S, Castrucci MR, et al. Molecular basis for the generation in pigs of influenza A viruses with pandemic potential. J Virol. 1998;72:7367–73. 9696833 10.1128/jvi.72.9.7367-7373.1998 PMC109961

[32] Yang H, Chen Y, Qiao C, Xu C, Yan M, Xin X, et al. Two different genotypes of H1N2 swine influenza virus isolated in northern China and their pathogenicity in animals. Vet Microbiol. 2015;175:224–31. 10.1016/j.vetmic.2014.11.031 25542286

[33] Lee JH, Pascua PNQ, Decano AG, Kim SM, Park S-J, Kwon H-I, et al. Evaluation of the zoonotic potential of a novel reassortant H1N2 swine influenza virus with gene constellation derived from multiple viral sources. Infect Genet Evol. 2015;34:378–93. 10.1016/j.meegid.2015.06.005 26051886

[34] Fobian K, Fabrizio TP, Yoon S-W, Hansen MS, Webby RJ, Larsen LE. New reassortant and enzootic European swine influenza viruses transmit efficiently through direct contact in the ferret model. J Gen Virol. 2015;96:1603–12. 10.1099/vir.0.000094 25701826 PMC4635450

[35] Nelson MI, Schaefer R, Gava D, Cantão ME, Ciacci-Zanella JR. Influenza A viruses of human origin in swine, Brazil. Emerg Infect Dis. 2015;21:1339–47. 10.3201/eid2108.141891 26196759 PMC4517702

[36] Peng X, Wu H, Xu L, Peng X, Cheng L, Jin C, et al. Molecular characterization of a novel reassortant H1N2 influenza virus containing genes from the 2009 pandemic human H1N1 virus in swine from eastern China. Virus Genes. 2016;52:405–10. 10.1007/s11262-016-1303-4 26980674

[37] Liu H, Tao J, Zhang P, Yin X, Ha Z, Zhang C. Pathogenic characteristics of a novel triple-reasserted H1N2 swine influenza virus. Biologicals. 2016;44:252–6. 10.1016/j.biologicals.2016.05.002 27230301

[38] Pascua PNQ, Song M-S, Lee JH, Baek YH, Kwon HI, Park S-J, et al. Virulence and transmissibility of H1N2 influenza virus in ferrets imply the continuing threat of triple-reassortant swine viruses. Proc Natl Acad Sci U S A. 2012;109:15900–5. 10.1073/pnas.1205576109 23019374 PMC3465388

