# Supplementary material for: Swine Influenza Virus (H1N2) Characterization and Transmission in Ferrets, Chile
Source: Emerg Infect Dis. 2017 Feb;23(2):241–51. doi: 10.3201/eid2302.161374 (PMC5324791; doi:10.3201/eid2302.161374)
Supplement: Technical Appendix — Phylogenetic trees of the internal genes of swine influenza virus (H1N2) from Chile, H1 virus α-2,6–linked sialic acid binding affinities, and H1 virus disease induction assessed by weight and temperature changes in infected ferrets. [file 16-1374-Techapp-s1.pdf]

Article DOI: http://dx.doi.org/10.3201/eid2302.161374

Swine Influenza Virus (H1N2) Characterization and Transmission in Ferrets, Chile

Technical Appendix

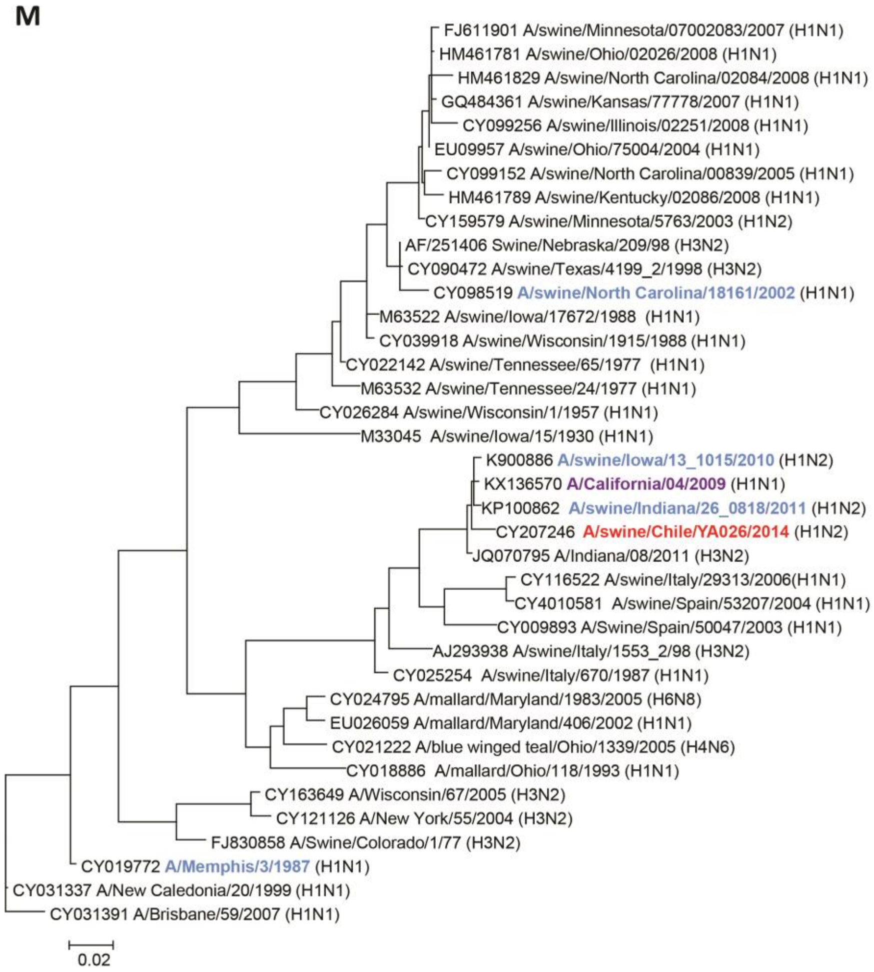

Technical Appendix Figure 1. Phylogenetic tree of the matrix (M) gene of Chilean H1N2 virus. Phylogenetic analysis was performed on complete M genome sequences using maximum likelihood (RAxML) and incorporating a general time-reversible model of nucleotide substitution with a gamma-distributed rate variation among sites. The Chilean H1N2 virus is shown in red, the A/California/04/2009 pandemic H1N1 virus is in purple and the swine viruses used as comparators throughout the studies are shown in blue. Scale bars represent the number of substitutions per site.

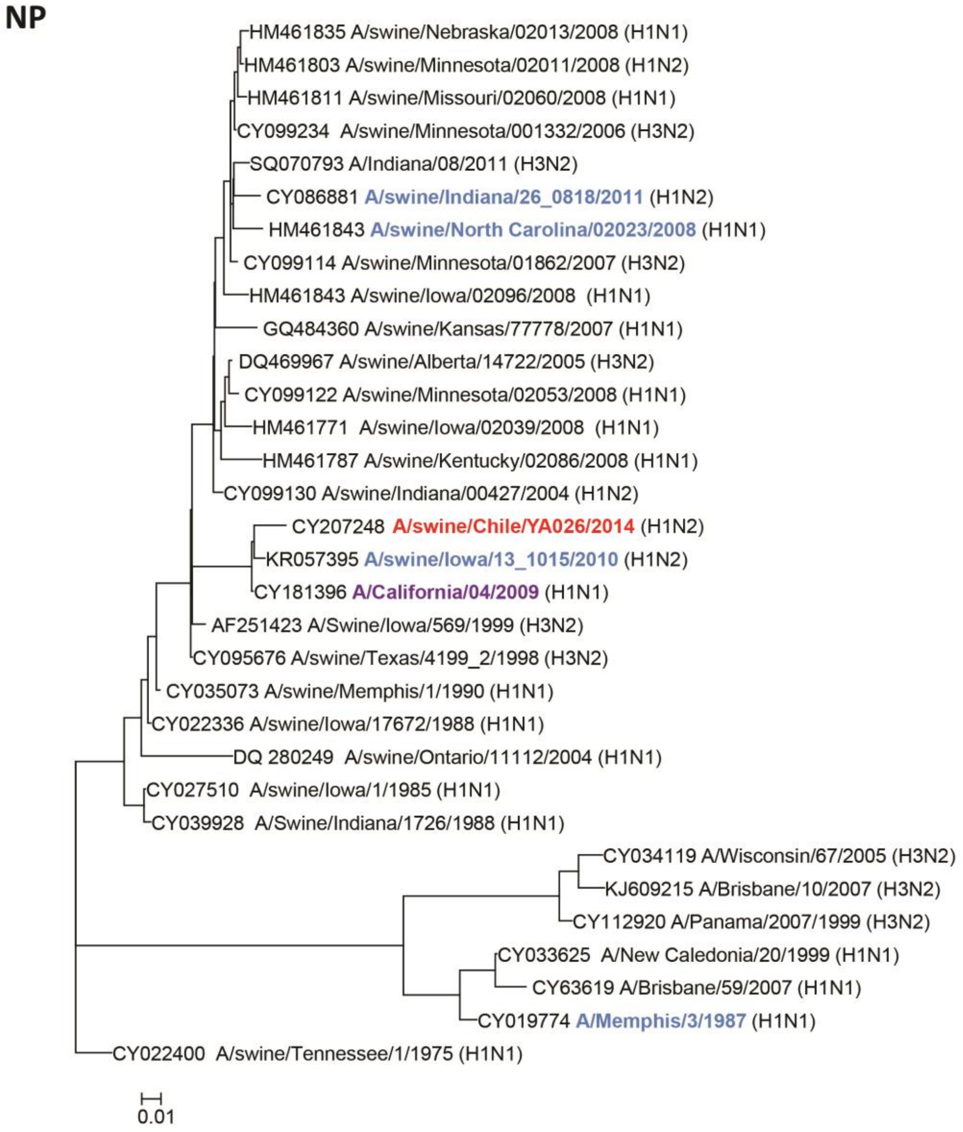

Technical Appendix Figure 2. Phylogenetic tree of the nucleoprotein (NP) gene of Chilean H1N2 virus. Phylogenetic analysis was performed on complete NP genome sequences using maximum likelihood (RAxML) and incorporating a general time-reversible model of nucleotide substitution with a gamma-distributed rate variation among sites. The Chilean H1N2 virus is shown in red, the A/California/04/2009 pandemic H1N1 virus is in purple and the swine viruses used as comparators throughout the studies are shown in blue. Scale bars represent the number of substitutions per site.

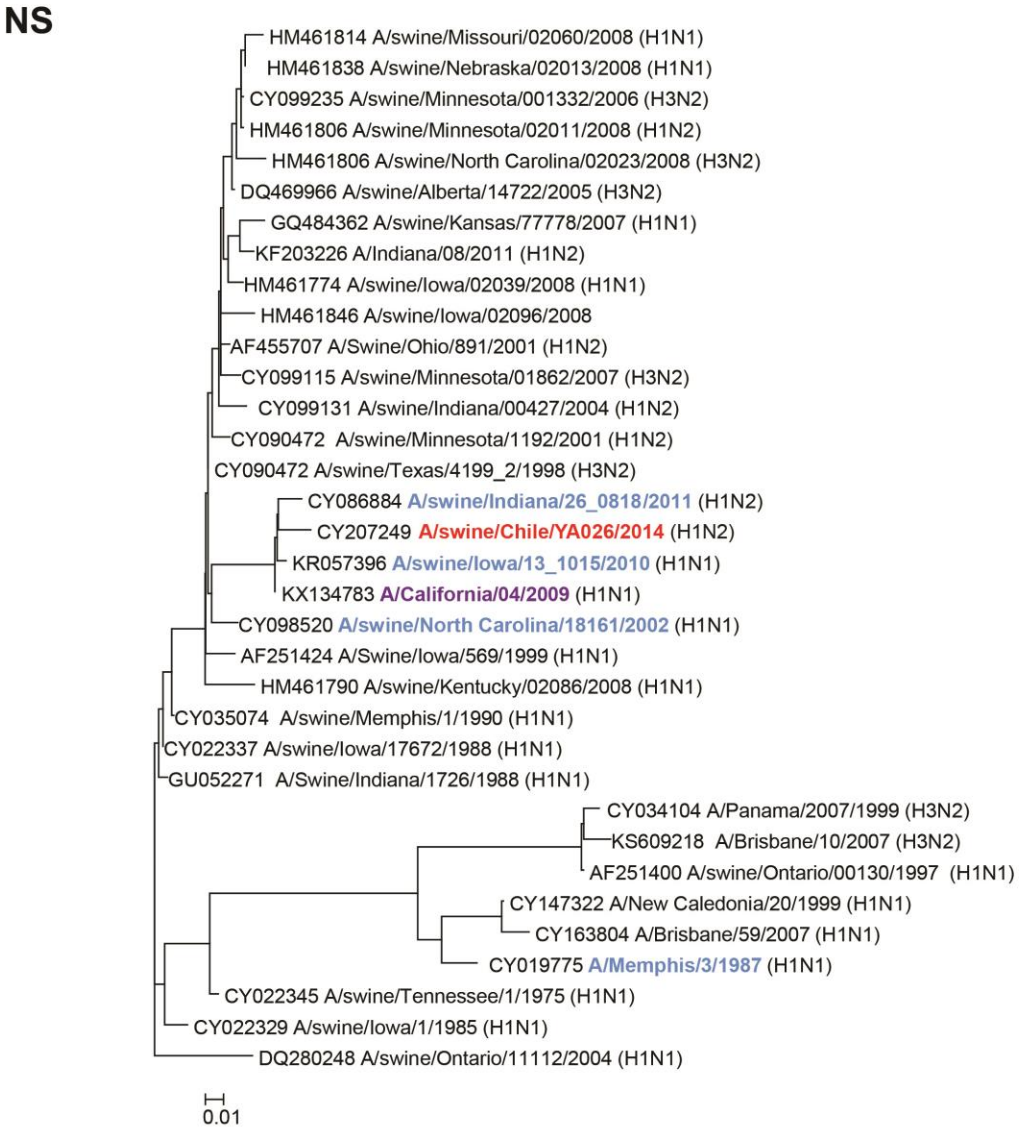

Technical Appendix Figure 3. Phylogenetic tree of the nonstructural (NS) gene of Chilean H1N2 virus. Phylogenetic analysis was performed on complete NS genome sequences using maximum likelihood (RAxML) and incorporating a general time-reversible model of nucleotide substitution with a gamma-distributed rate variation among sites. The Chilean H1N2 virus is shown in red, the A/California/04/2009 pandemic H1N1 virus is in purple and the swine viruses used as comparators throughout the studies are shown in blue. Scale bars represent the number of substitutions per site.

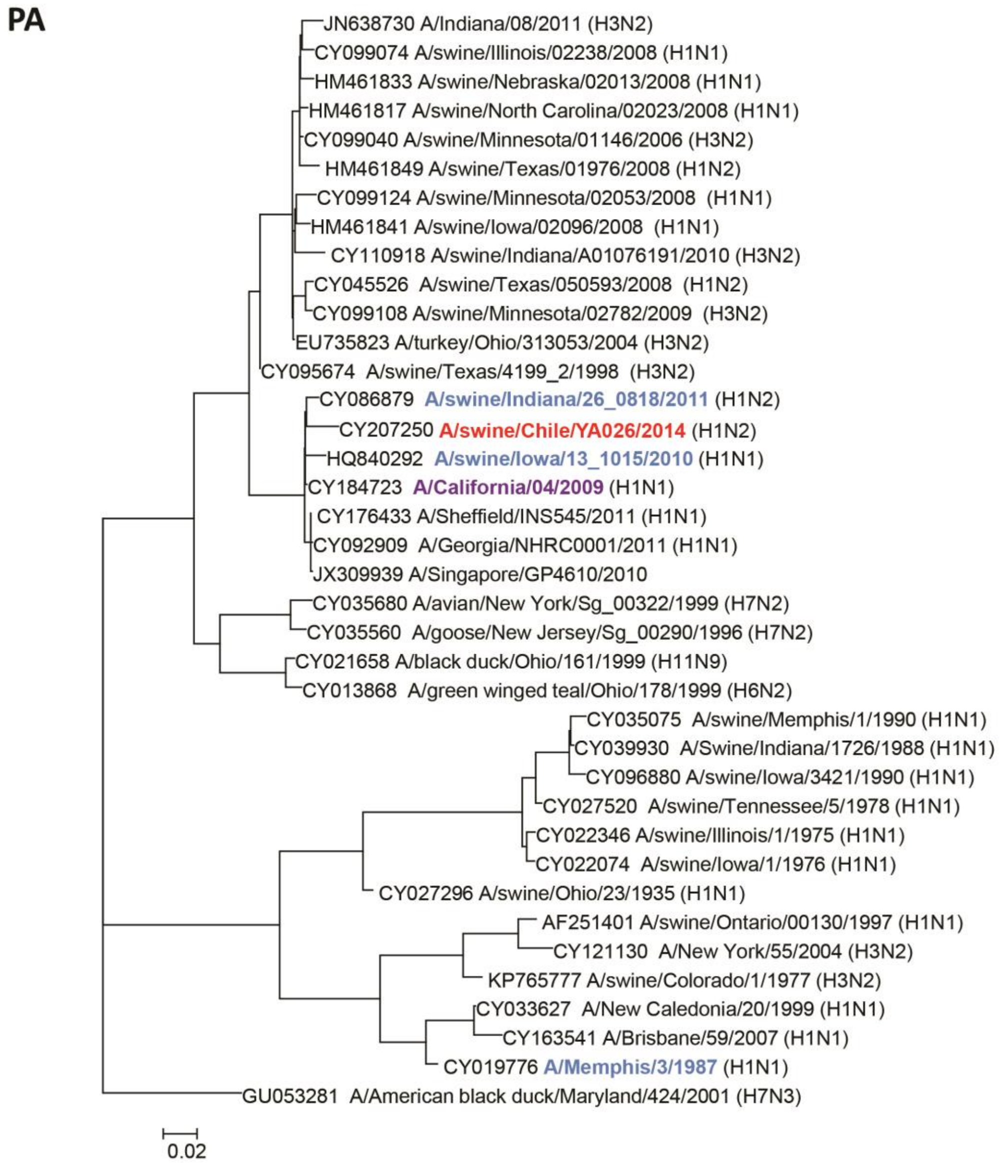

Technical Appendix Figure 4. Phylogenetic tree of the polymerase acid (PA) gene of Chilean H1N2 Phylogenetic analysis was performed on complete PA genome sequences using maximum likelihood (RAxML) and incorporating a general time-reversible model of nucleotide substitution with a gamma-distributed rate variation among sites. The Chilean H1N2 virus is shown in red, the A/California/04/2009 pandemic H1N1 virus is in purple and the swine viruses used as comparators throughout the studies are shown in blue. Scale bars represent the number of substitutions per site.

PB1

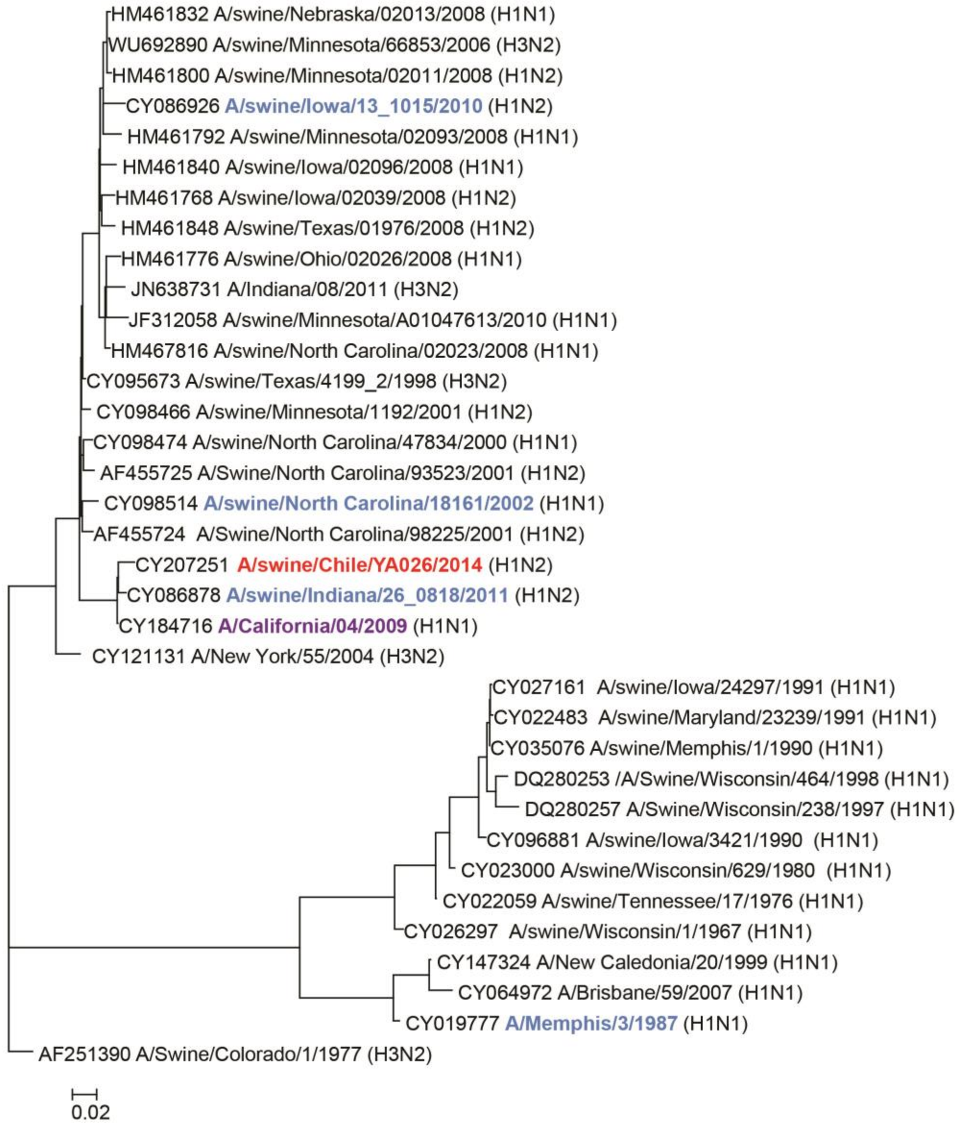

Technical Appendix Figure 5. Phylogenetic tree of the polymerase basic 1 (PB1) gene of Chilean H1N2 virus. Phylogenetic analysis was performed on complete PB1 genome sequences using maximum likelihood (RAxML) and incorporating a general time-reversible model of nucleotide substitution with a gamma-distributed rate variation among sites. The Chilean H1N2 virus is shown in red, the A/California/04/2009 pandemic H1N1 virus is in purple and the swine viruses used as comparators throughout the studies are shown in blue. Scale bars represent the number of substitutions per site.

PB2

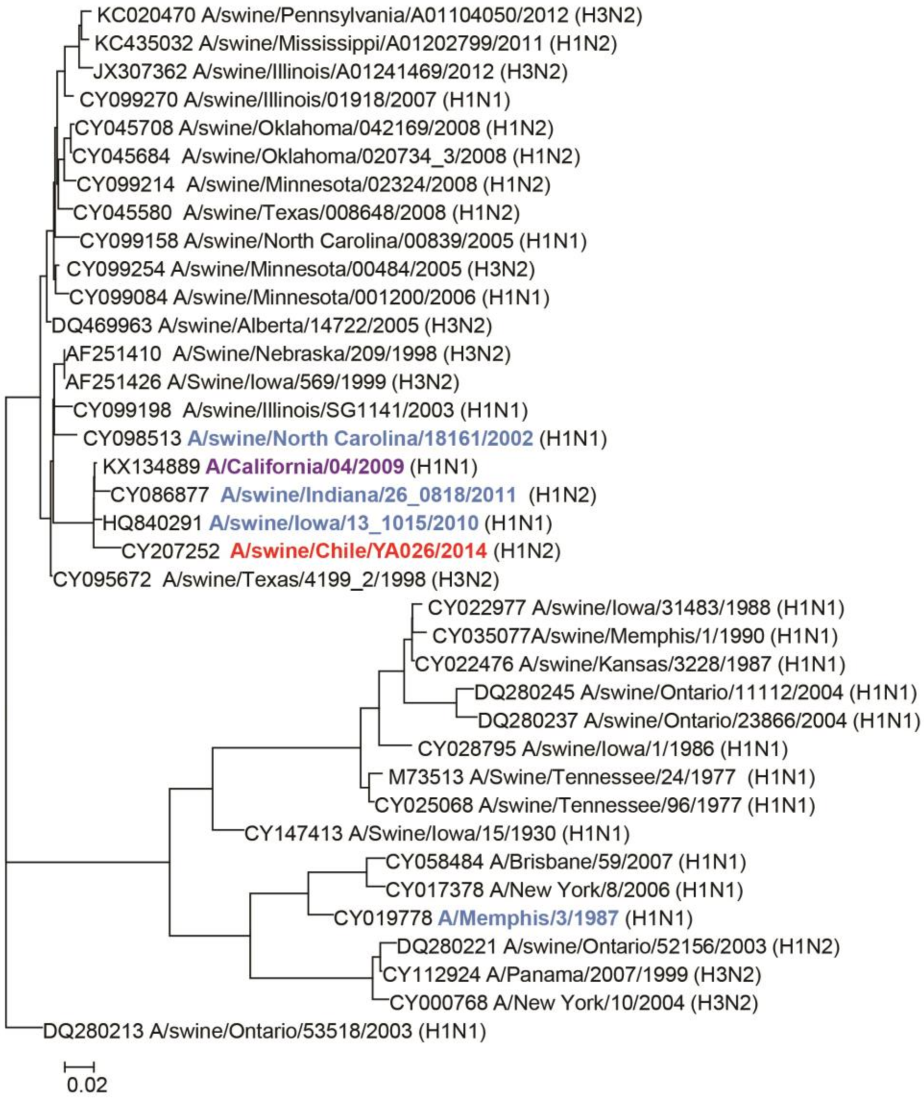

Technical Appendix Figure 6. Phylogenetic tree of the polymerase basic 2 (PB2) gene of Chilean H1N2 virus. Phylogenetic analysis was performed on complete PB2 genome sequences using maximum likelihood (RAxML) and incorporating a general time-reversible model of nucleotide substitution with a gamma-distributed rate variation among sites. The Chilean H1N2 virus is shown in red, the A/California/04/2009 pandemic H1N1 virus is in purple and the swine viruses used as comparators throughout the studies are shown in blue. Scale bars represent the number of substitutions per site.

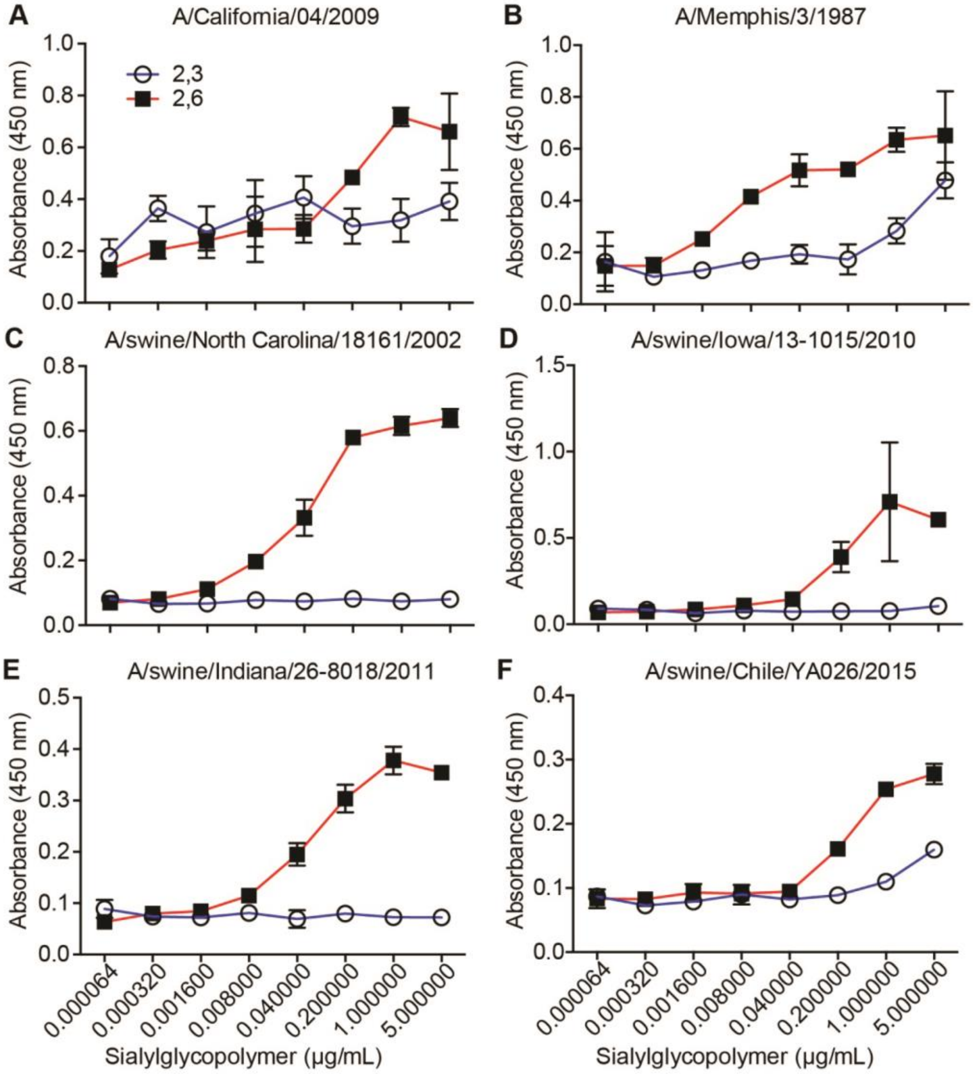

Technical Appendix Figure 7. Sialic acid–binding affinities of H1 viruses. Both human (A-B) and swine (C-F) H1 viruses had increased binding to α-2,6–linked sialic acids suggesting increased mammalian binding efficiency. Data are representative of 3 replicates per virus. Data is presented as mean ± SEM.

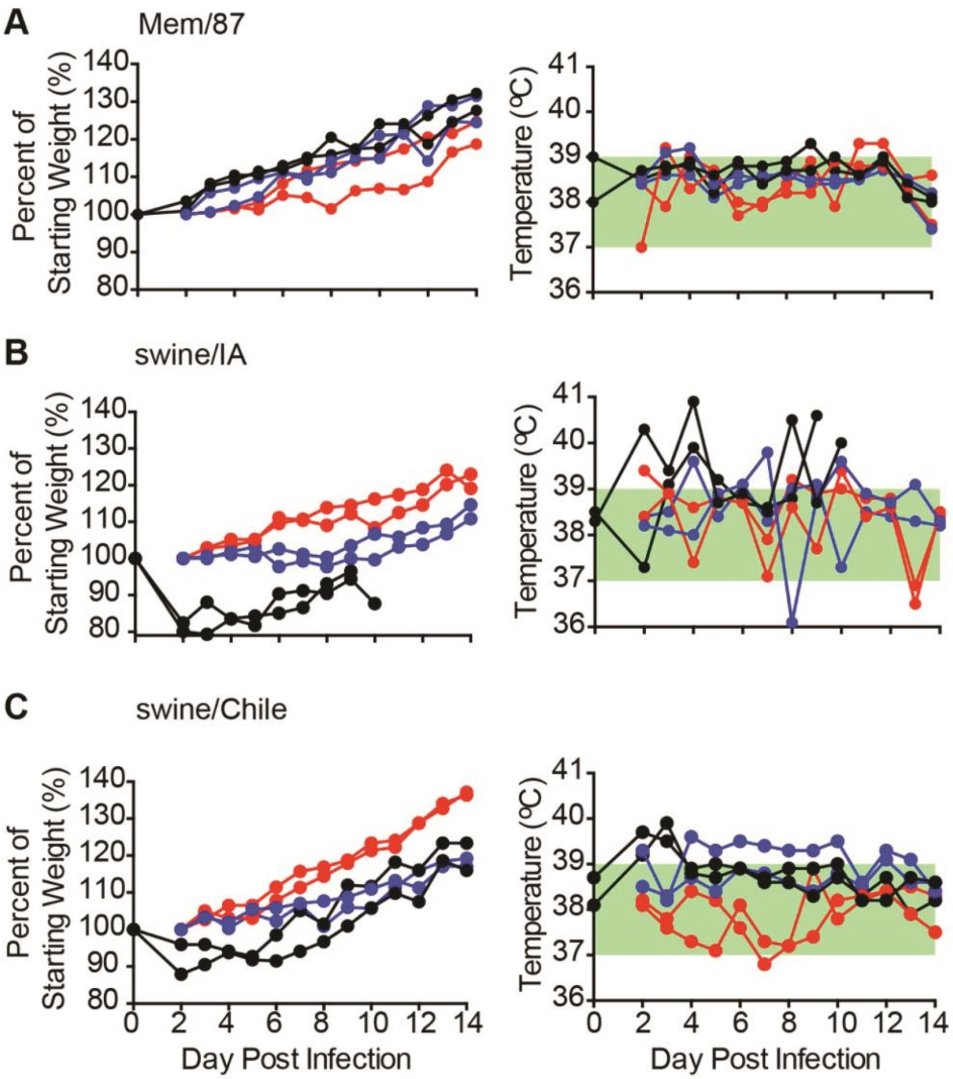

Technical Appendix Figure 8. Weight and temperature during ferret transmission of H1 viruses. Ferrets (n = 2/group) were inoculated with 10^6 units of H1 influenza virus (black lines). Naive ferrets (n = 2) were placed either in the same cage with the infected group (direct contact, blue lines) or housed in separate cages (respiratory transmission, red lines) and weight and temperature data was collected every 24 hours postinfection for (A) Mem/87, (B) swine/IA, and (C) swine/Chile viruses. Lines represent individual animals. Green shading indicates normal temperature range for ferrets (http://weaselwords.com/ferret-facts/).
